# A new species of *Gastrosaccus* Norman, 1868 (Mysida, Mysidae, Gastrosaccinae) from a sandy shore of Indonesia

**DOI:** 10.3897/zookeys.438.8031

**Published:** 2014-09-01

**Authors:** Yukio Hanamura, Rose O.S.E. Mantiri, Susumu Ohtsuka

**Affiliations:** 1 National Research Institute of Fisheries Science, Fuku-ura 2−12−4, Kanazawa-ku, Yokohama 236−8648, Japan; 2 Faculty of Fisheries and Marine Science, University of Sam Ratulangi, Manado, North Sulawesi, Indonesia; 3 Takehara Marine Science Station, Setouchi Field Science Center, Hiroshima University, Minato-machi 5−8−1, Takehara, Hiroshima Prefecture 725−0024, Japan

**Keywords:** Mysid shrimp, taxonomy, new species, Lombok Island, Indonesia

## Abstract

A new species of the mysid crustacean genus *Gastrosaccus* Norman, 1868 (Mysida, Mysidae, Gastrosaccinae) is reported from a sandy shore of Lombok Island, Indonesia. These specimens resemble *G. sorrentoensis* Wooldridge & McLachlan, 1986 and *G. yuyu* Bamber and Morton, 2012 by the possession of an articulated process on the fifth abdominal somite together with a fringe of spine-like filaments on the posterodorsal margin of the carapace. The Lombok population differs from the known congeners by having comparatively fewer numbers of carpopropod segments on the endopod of the third to eighth thoracic limbs and the conformation in the telson and in the male third pleopod. Hence, *G. lombokiensis* **sp. n.** is proposed herein as a third species of “*G. sorrentoensis*” species group.

## Introduction

The mysid genus *Gastrosaccus* Norman, 1868 (Crustacea, Mysida) currently comprises 24 species (Mees and Meland 2013) and has been recorded in the coastal waters of the eastern Atlantic to the western Pacific, through the Indian Ocean. Among these members, two species are particularly remarkable by having an articulated broad process on the fifth abdominal somite as well as a fringe of spine-like filaments on the posterodorsal margin of the carapace, i.e., *Gastrosaccus sorrentoensis* Wooldrige & McLachlan, 1986 recorded from Western Australia and *Gastrosaccus yuyu* Bamber & Morton, 2012 from Java Island, Indonesia. They are assigned in this paper to the “*Gastrosaccus sorrentoensis*” species group. Interestingly, *Gastrosaccus yuyu* is reported to be harvested for human consumption on sandy beaches of Java (Bamber and Morton 2012).

During a recent survey of coastal crustaceans in Indonesia, which aimed in part to clarify the nature of planktonic crustacean fisheries in the country (Mantiri et al. 2012), several specimens of gastrosaccini mysids sharing the aforementioned intriguing characters were found from a sandy beach collection in Lombok Island. Morphological analysis revealed that these specimens differ from the known relatives in having the carpopropodus of the thoracic endopods with comparatively fewer numbers of segments, as well as the ornamentation in the telson and the structure of the third male pleopod. Consequently, *Gastrosaccus lombokiensis* sp. n. is described here as the third species of the “*Gastrosaccus sorrentoensis*” species group from Lombok Island, southern Indonesia.

## Materials and methods

The sampling site, Padak Guar beach is located on the north-east coast of Lombok Island, Indonesia. The beach substratum is predominately fine sand particles.

The material used in this paper was collected by the second author (RM) using a push net (2 m in mouth width, 3 m long, and 1 mm mesh openings), which was towed along the beach on the evening of June 6^th^, 2010. Mysid specimens were fixed in 70−99% ethanol immediately upon collection and preserved prior to analysis.

Body size of mysids (TL: mm) was measured between the distance from the apex of the rostrum to the posterior end of the telson excluding the apical spine-like setae. Description is based on adult specimens unless stated otherwise. Watling’s (1989) setal/spine system was basically adopted for the terminology of cuticle projections.

The type specimens are deposited in the National Museum of Nature and Science at Tsukuba, Japan (NSMT).

## Taxonomy

### Family Mysidae Haworth, 1825
Subfamily Gastrosaccinae Norman, 1892

#### Genus Gastrosaccus

Taxon classificationAnimaliaMysidaMysidae

Norman, 1868

Gastrosaccus Norman, 1868: 438.
Acanthocaris Sim, 1872: 185.
Pontomysis Czerniavsky, 1882: 77.

##### Type species.

*Mysis spinifera* Goës, 1864; by monotypy.

##### Remarks.

Norman (1868) instituted the genus *Gastrosaccus* to accommodate mysid specimens having a characteristic formation of marsupium, for which the pleura of the female first abdominal somite are greatly expanding as to support the oostegites. When establishing *Gastrosaccus*, Norman actually intended to place *Mysis spinifera* Goës, 1864, in the genus but he mistakenly referred his specimens to *Mysis sancta* van Beneden, 1861 because he first thought that *Mysis spinifera* could be a synonym of *Mysis sancta*. This was later corrected by replacing it with *Gastrosaccus spinifer* (see Norman 1892, Tattersall and Tattersall 1951). The genus currently contains 24 species (Mees and Meland 2013).

#### Gastrosaccus lombokiensis sp. n.

Taxon classificationAnimaliaMysidaMysidae

http://zoobank.org/69425BB7-03E6-4FA1-8AF9-18A878A4CC7B

**Figure 1.**
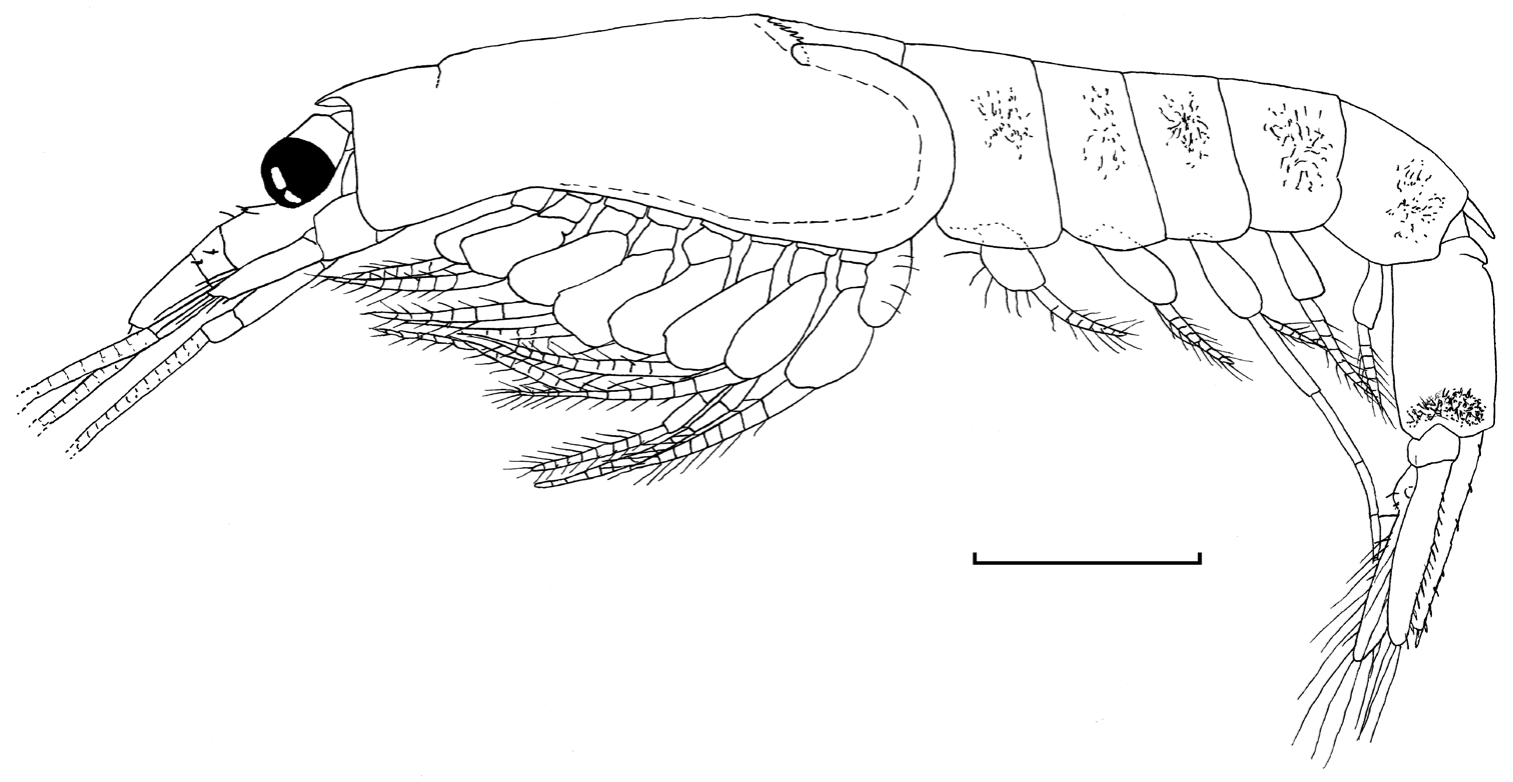
*Gastrosaccus lombokiensis* sp. n., entire body of paratype male (TL 6.9 mm) in lateral view. Scale = 1 mm.

Figs 1 [Fig F2] [Fig F3] [Fig F4] –5

##### Holotype.

Male (TL ca. 7.5 mm) (NSMT−Cr 22940), sandy beach, Padak Guar (08°25.665'S, 116°42.561'E), Lombok Island, push net, evening time (17:00), 6 June 2010, coll. R. Mantiri.

##### Paratypes.

11 males (TL 4.0−ca. 7.5 mm), 3 females (TL 4.0−ca.7.5 mm), 3 juvs (TL 2.8–3.5 mm) (NSMT−Cr 22941), data same as for holotype.

##### Diagnosis.

Rostrum produced into sub-triangular fig. Posterodorsal edge of carapace bearing 7−12, commonly 9−11, spine-like filaments between dorsolateral slits and normally further 5−10 feeble denticulations/undulations on each side of posterolateral lobe posterior to slit. Fifth somite of abdomen with articulated triangular process at posteromedian end; pleuron of female first somite greatly expanded, fully covering marsupium. Telson typical of Gastrosaccinae form, with apical cleft occupying 1/8−1/6 length of telson, armed laterally with 8 or 9 robust spine-like setae, including posteriormost one. Uropod with exopod shorter than endopod, armed laterally with 13 or 14 spine-like setae; endopod with 6 or 7 spine-like setae on mesial margin. Labrum with single anteromedian tooth and additional smaller spines absent. Third pleopod of male with endopod multi-articulated; exopod greatly elongated, extending well beyond sixth abdominal somite, comprising 4 major segments, and basal segment without distinct sub-articles, distal segment 0.65–0.7 times length of penultimate one, armed with short sub-terminal seta and moderately long 2 terminal setae. First pleopod of female bi-lobed, second to fifth pleopods rudimentary, unsegmented lobe.

##### Description.

Male. Body (Fig. 1) moderately robust.

**Figure 2. F2:**
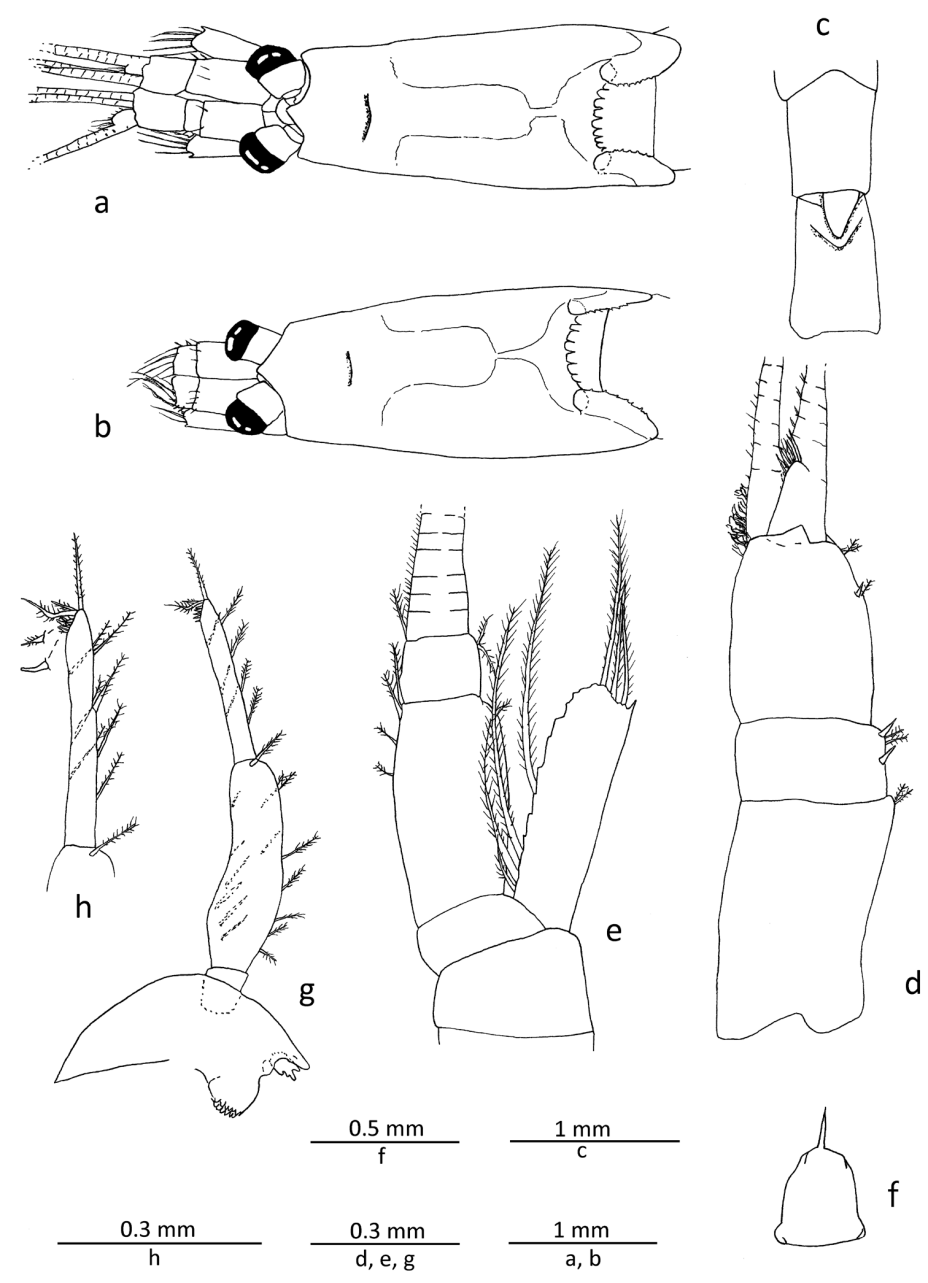
*Gastrosaccus lombokiensis* sp. n., paratype male (TL ca. 7.5 mm) (**a, c−h**) and paratype female (TL ca. 7.5 mm) (**b**): **a** carapace and cephalic appendages in male, dorsal view **b** same in female **c** fifth and sixth abdominal somites, dorsal view **d** right antennule, dorsal view **e** right antenna, dorsal view **f** labrum, ventral view **g** right mandible, external view **h** same, distal article of mandibular palp enlarged.

Carapace (Figs 1, 2a, b) produced into sub-triangular rostrum with sub-acute apex; posterior dorsal margin deeply excavate, leaving exposed last thoracic somite, dorsolateral part of emargination forming slit with posterior lobe overlapping onto anterior one; lateral wing of carapace well developed, extending to anterior 1/3 of first abdominal pleuron; anterolateral part rounded; cervical sulcus marked dorsally anterior to anterior 1/3; posterior dorsal edge between dorsal slits bearing 7−12, most commonly 9−11, spine-like filaments, and further 5−10 feeble denticles/undulations on each margin posterior to slit (often hard to define exact numbers due to its poor development particularly in posterior part).

Abdomen (Figs 1, 2c) with anterior 4 somites rounded dorsally, sub-equal in length; fifth somite rounded dorsally, 1.3−1.4 times longer than precedents, with remarkable articulated sub-triangular process on posterodorsal margin; sixth somite very slightly longer than fifth, with transverse carinate ridge on anterodorsal part; brown to dark-brown melanophores on lateral surface of first to fifth somites and also in posterolateral part of sixth somite in ethanol preserved specimens.

Antennule (Fig. 2d) with basal segment longest, very slightly shorter than length of 2 anterior segments combined; second segment shortest, about 0.55 times longer than wide, with 2 spine-like setae, one at mid-length and another one at anterior end of lateral margin; third segment about twice the length of second, with short spine-like seta at distal 1/3 of lateral margin; lateral flagellum slightly widened at mesiobasal part, forming somewhat small male lobe bearing moderately long sensory setae; mesial flagellum more slender than lateral one. Antenna (Fig. 2e) with scale proportionately short, barely reaching or falling slightly short of anterior end of second antennular segment, slightly more than 3 times as long as wide, lateral margin smooth, distolateral spine not extending beyond anterior margin of lamella; antennal peduncle long, extending slightly beyond scale, penultimate segment elongated, approximately 3 times as long as distal one.

Labrum (Fig. 2f) with single sharp anteromedian tooth, additional teeth absent.

**Figure 3. F3:**
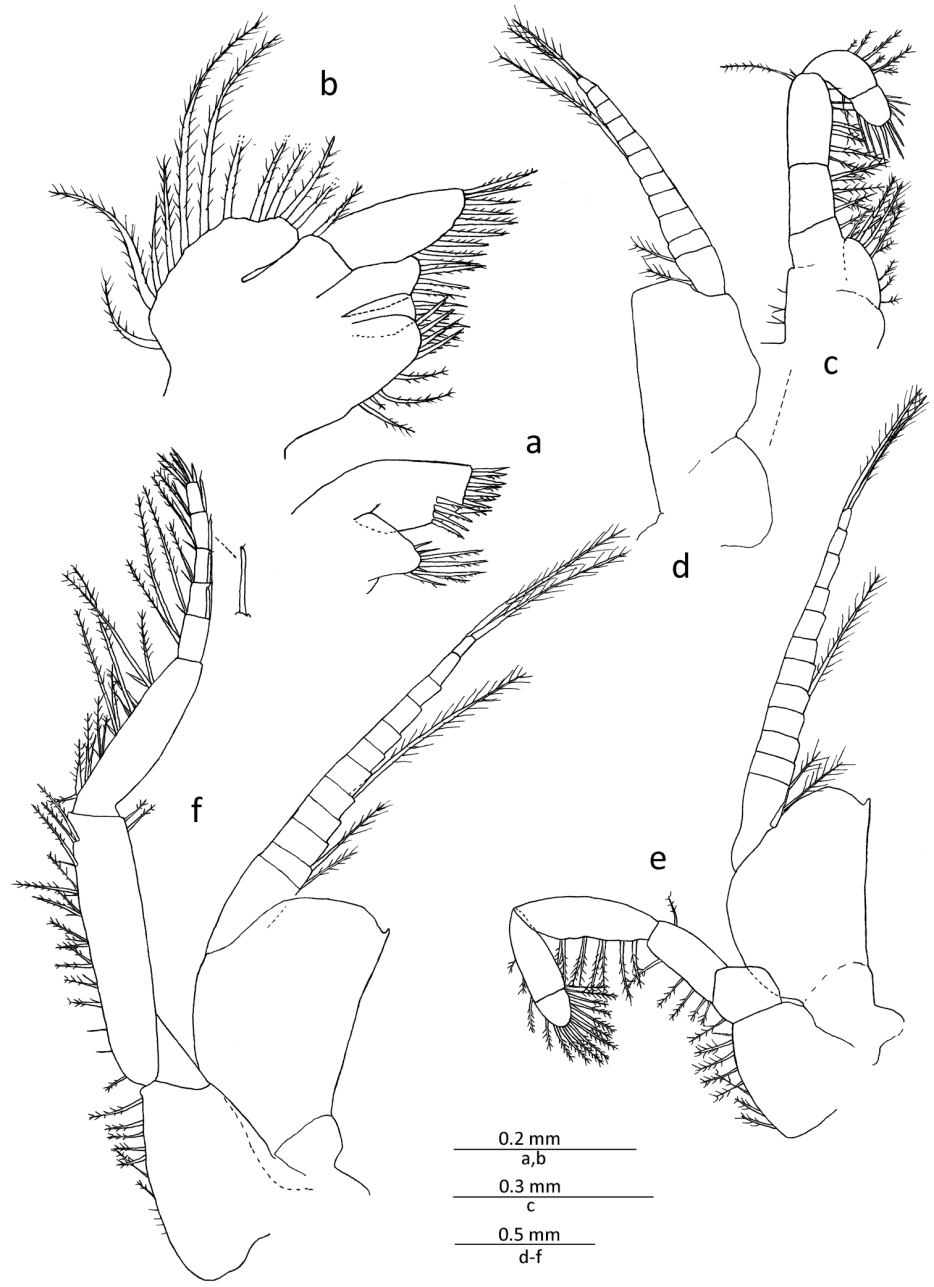
*Gastrosaccus lombokiensis* sp. n., paratype male (TL ca. 7.5 mm): **a** right maxillule, external view **b** right maxilla, external view **c** right endopod of first thoracic limb, external view **d** right exopod of first thoracic limb, external view **e** right second thoracic limb, internal view **f** right third thoracic limb, posterior view.

Mandibular palp (Fig. 2g, h) composed of 3 segments, distal segment about 0.8 times length of penultimate, with 2 long terminal setae, of which proximal one curved around its mid-length, often forming sub-chelate structure with terminal segment, and another seta moderately long at distal end, and also about 3 short spine-like setae as well as several short obtuse setae, all confined to distal part, forming comb-like structure. Mouthparts (maxillule, maxilla and second thoracopod) general form of genus (Fig. 3a, b, e). First thoracopod (Fig. 3c, d) with basal fig of exopod about 1.6 times length of its width.

**Figure 4. F4:**
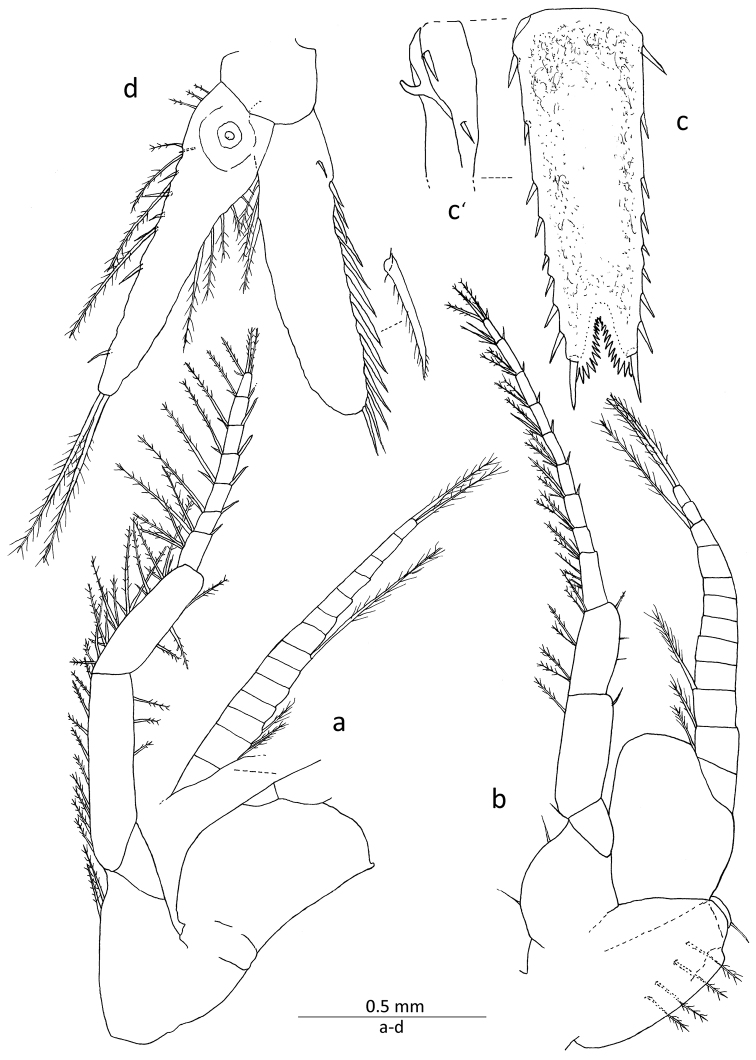
*Gastrosaccus lombokiensis* sp. n., paratype male (TL ca. 7.5 mm): **a** right sixth thoracic limb, posterior view **b** left eighth thoracic limb and penis, anterior view **c** telson, dorsal view **c**’ basal part of telson, lateral view **d** right uropod, dorsal view.

Third to eighth thoracopods (Figs 3f, 4a, b) similar in basic structure; exopods with basal fig smooth on lateral margin, and small process at distolateral corner except for eighth limb, and also with 11–14 segmented flagellar part; third to eighth endopods with carpopropodus composed of 5−9 segments, progressively increasing in numbers posteriorly, in which eighth endopod is divided into 8 or 9 segments.

Penis (Fig. 4b) about 1.7 times as long as wide, with short terminal seta directed posteriorly and 4 or 5 long setae along lateral surface.

**Figure 5.**
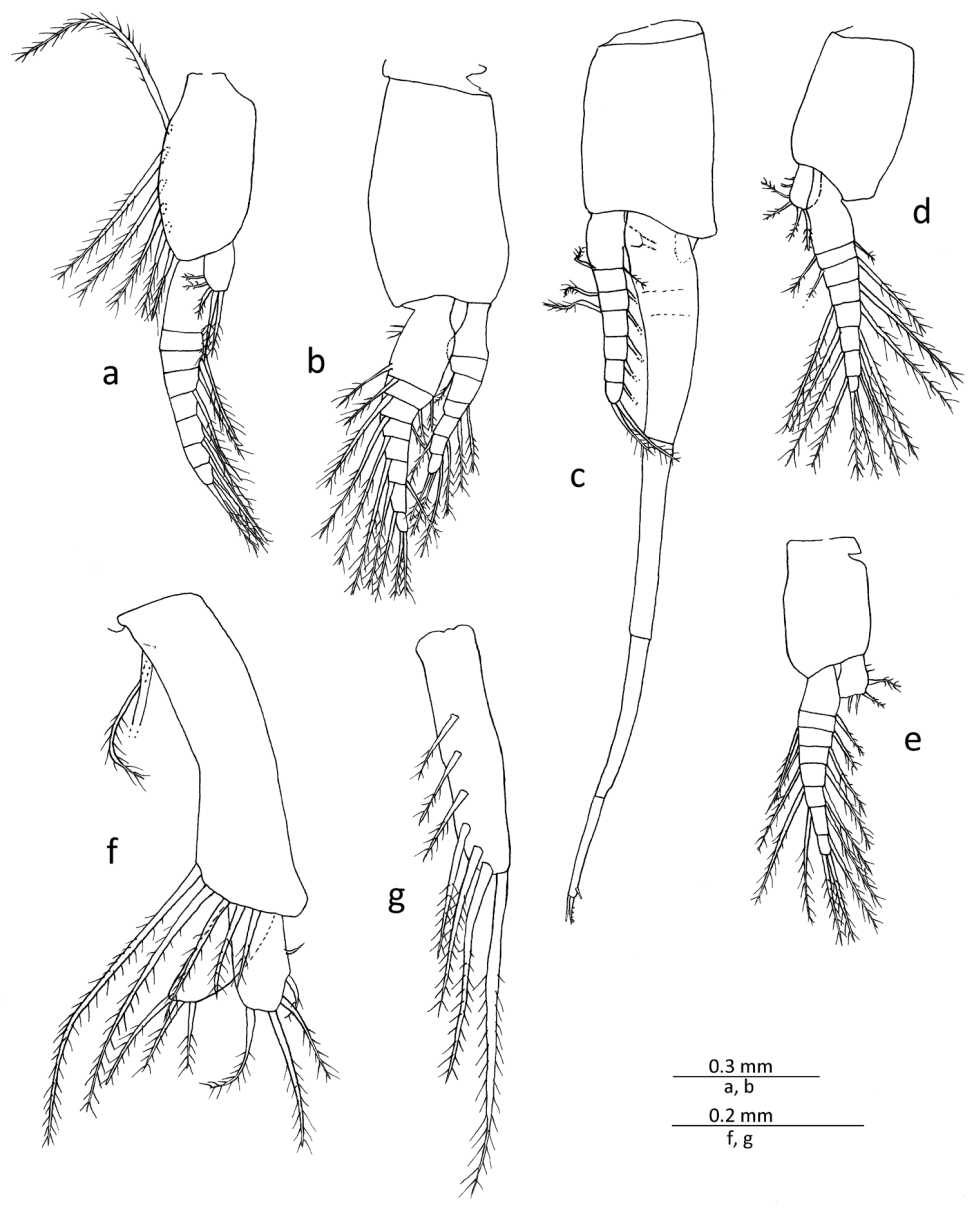
*Gastrosaccus lombokiensis* sp. n., paratype male (TL ca. 7.5 mm) (**a−e**) and paratype female (TL ca. 7.5 mm) (**f, g**): **a** right male first pleopod, mesial view **b** right male second pleopod, mesial view **c** right male third pleopod, lateral view **d** right male fourth pleopod, lateral view **e** right male fifth pleopod, mesial view **f** right first female pleopod, mesial view **g** right female third pleopod, mesial view.

All pleopods of male biramous (Figs 1, 5a–e). First pleopod with sympod bearing several long setae along anterior margin; endopod rudimentary, unsegmented lobe possessing several marginal setae distally; exopod articulated to about 8 segments. Second pleopod with endopod articulated to about 7 segments; exopod articulated to about 8 segments, slightly longer than endopod. Third pleopod with endopod articulated to 6 or 7 segments; exopod greatly elongated, extending beyond posterior end of abdomen fully by distal segment, composed of 4 major segments, basal segment markedly compressed and broader than distal series of segments, sub-equal length or very slightly longer than second one, without distinct sub-segments but with slight indication of 2 incipient articulations, second segment tubular and unarmed, penultimate segment bearing short spine-like sub-distal seta, distal segment shortest, 0.65−0.70 times length of penultimate, armed distally with 2 moderately long spine-like setae with subsidiary setules on its distal half. Fourth and fifth pleopods similar in basic form, endopod unsegmented, bearing a few setae on distal margin; exopod articulated to about 8 segments.

Uropod (Fig. 4d) with exopod shorter than endopod, lateral margin armed with 13 or 14 robust spine-like setae, length increasing posteriorly, each spine-like seta with subsidiary setules on posterior margin and also short terminal protrusion; endopod extending beyond telson and reaching as far as end of distal telson setae, commonly bearing 7, occasionally 6, spine-like setae along entire mesial margin, showing general tendency towards increased setae length distally.

Telson (Fig. 4c) 1.1–1.2 times as long as sixth abdominal somite and 2.6−2.8 times as long as wide, apical cleft occupying about 1/7 length of telson, bearing approximately 15 dentations along each mesial margin; lateral margin with 8 or 9 spine-like setae of varying length, including longest apical seta, penultimate seta distinctly shorter than apical and located noticeably posterior to level of anterior edge of apical cleft, proximal lateral seta sub-equal in length to penultimate one, posterior series of lateral setae spaced equidistantly and unarmed gap between apical and penultimate setae subequal in distance with those of others; procurved sharp process (Fig. 4c’) present at proximal part of ventral side. Melanophores widely distiributed on dorsal surface of telson but not along mesiodorsal axis and often distal part of apical lobes in both sexes.

Female. Sexual dimorphism as common for genus.

Pleuron of first abdominal somite greatly expanded, fully covering marsupium.

Antennule (Fig. 2b) might differ slightly from that of male but is damaged in the present material and not dissected.

First pleopod (Fig. 5f) biramous, with unsegmented endopod and exopod, sub-equal in length and markedly shorter than sympod, exopod and endopod each with several long plumose setae on distal part; sympod with a few long setae near anterobasal part and further several long setae along anterior distal margin. Second to fifth pleopods (Fig. 5g) similar in shape and size, all rudimentary, unarticulated rod-shaped lobe, bearing several long setae on lateral surface and also on distal margin.

##### Etymology.

The name “*lombokiensis*” refers to the type locality, Lombok Island.

##### Remarks.

This species is remarkable among the members of *Gastrosaccus* in having an articulated process at the posterodorsal end of the fifth abdominal somite throughout its life stages, coupled with a fringe of spine-like filaments on the posterodorsal margin of the carapace. The combination of these intriguing characters is shared by *Gastrosaccus sorrentoensis* Wooldridge & McLachlan, 1986 from Western Australia (see also Hanamura 1998) and *Gastrosaccus yuyu* Bamber & Morton, 2012 from Java Island, Indonesia.

Specimens from Lombok Island have the carpopropodus of the thoracic endopods comprising fewer numbers of segments, and at most 8 or 9 segments in the eighth limb compared to 13 or more segments in the previously known species (Table 1). There is a slight possibility that the paucity in the segment counts in Lombok specimens is due to comparatively smaller body sizes, thus showing a condition of younger stages. Although ovigerous females were not available in our collection, the largest female has a well-developed first abdominal pleuron, which is fully covering the marsupium lamellae. Also larger males (> ca. 6.5 mm) were considered as adult because there third pleopods are fully developed, extending well beyond the posterior margin of the abdomen at least by length of distal article. Furthermore, the posterior part of the *vas deferens* of several males exhibited whitish colour just above the ejaculatory duct, common in mature males.

**Table 1.** Comparison of the “*Gastrosaccus sorrentoensis* Wooldridge & McLachlans, 1986” species group.

Item/species	*Gastrosaccus sorrentoensis* Wooldridge & McLachlan, 1986	*Gastrosaccus yuyu* Bamber & Morton, 2012	*Gastrosaccus lombokiensis* sp. n.
Rostrum	wide triangular protrusion with sub-acute apex	tongue-like protrusion with widely round apex	sub-triangular protrusion with sub-acute apex
Posterior margin of carapace	about 25 spine-like filaments between dorsolateral slits and 14–25 filaments or undulations on dorsolateral edge posterior to slits (fewer number of filaments in smaller specimens)	6–12 spine-like filaments between dorsolateral slits and 6–12 filaments on dorsolateral edge posterior to slits	7–12, commonly 9–11, spine-like filaments between dorsolateral slits and 5–10 very feable denticles or undulations on dorsolateral edge posterior to slits
Mandibular palp	distal segment 0.8 times length of penultimate, terminally with long slender seta and long robust undulate seta directed laterally	distal segment 0.9 times length of penultimate, terminally with 2 slender setae not distinctly directed laterally	distal segment 0.8 times length of penultimate, terminally with long slender seta, long undulate seta directed laterally and one moderately long seta
First thoracic limb	basal fig of exopod about twice length of width	basal fig of exopod about 1.5 times length of width	basal fig of exopod about 1.6 times length of width
Third to eighth thoracic limbs	carpopropodus of endopod with 8–16 segments, and 14–16 on eighth	carpopropodus of endopod with 12–14 segments, and probably 13 or 14 on eighth	carpopropodus of endopod with 5–9 segments, and 8 or 9 on eighth
Female first pleopod	endopod slightly longer than exopod; sympod about 4 times length of width	endopod distinctly longer than exopod; sympod about 4 times length of width	endopod slightly longer than exopod; sympod about 3 times length of width
Male third pleopodal exopod	basal segment noticeably longer than second, divided into distinct 3 short sub-segments; distal segment 0.75 times length of penultimate	basal segment noticeably longer than second, divided into 2 or 3 sub-segments; distal segment 0.8 times length of penultimate	basal segment not divided into distinct sub-segments but with slight sign of possible 2 indicipent segmentations; distal segment 0.65–0.70 times length of penultimate
Uropodal endopod	5 or 6 irregularly spaced mesial setae along entire margin	7 or 8 irregularly spaced mesial setae confined to basal 2/3	usually 7, occasionally 6, irregularly spaced mesial setae along entire margin
Telson	sharp process present at proximalventral part; about 2.5 times as long as wide; apical cleft 1/6 length of telson; 7 or 8 robust lateral setae, penultimate seta arising around level of anterior end of apical cleft, apical spine distinctly longer than penultimate	sharp process absent at proximal ventral part; about 2.8 times as long as wide, apical cleft 1/8 length of telson; 10 robust lateral setae, penultimate seta arising anterior to anterior end of apical cleft, apical spine very slightly longer than penultimate	sharp procurved process present at proximal ventral part, 2.6–2.8 times as long as wide, apical cleft 1/8–1/7 length of telson; 8 or 9 robust lateral setae, penultimate seta arising distinctly posterior to anterior end of apical cleft, apical spine distinctly longer than penultimate
Body size	male 7.0–9.3 mm, female 8.5–11.3 mm	male holotype 8.4 mm, female 9–13 mm	male ca. 6.5–7.5 mm, female ca. 7.5 mm
Occurrence	surf zone of sandy beach, Perth, Western Australia	surf zone of sandy beach, Java, Indonesia	surf zone of sandy beach, Lombok, Indonesia
Data source	Wooldridge and McLachlan 1986; Bamberand Morton 2012	Bamber and Morton 2012	present study

The male third pleopodal exopod of this group basically has four major segments. In *Gastrosaccus sorrentoensis* and *Gastrosaccus yuyu*, the basal article is divided further into two or three short sub-segments instead of an entire basal article, without distinct short sub-segments. In the Lombok population, we do not find distinct short sub-articulations. However, a slight indication of incipient sub-segmentations was observed in the internal muscular part; but an associated segmented exoskeleton could not be found. Also the shape of the telson is noticeably different among the three populations and these features are regarded as size independent.

Compared to those of *Gastrosaccus sorrentoensis*, the Lombok population tended to have larger number of spine-like setae in the uropodal endopod and telson despite their smaller body size. The female telson of *Gastrosaccus sorrentoensis* bears a pair of pigmented spots at the inner side of the fifth lateral setae (Wooldridge and McLachlan 1986) whereas in Lombok specimens the telson is tinted by melanophores widely along the margins in both sexes.

According to Bamber and Morton (2012), the Javanese *Gastrosaccus yuyu* is devoid of an anteroventral process on the telson, and this feature is unique among the three populations.

Our observation suggested that *Gastrosaccus lombokensis* starts to develop external secondary sexual characteristics at a size around TL 3.5−4 mm in both sexes.

##### Distribution.

This species is known only from the sandy shore of Lombok Island, Indonesia.

### Further notes on morphological characteristics of *Gastrosaccus* and related genera

The possession of an articulated process on the fifth abdominal somite together with spine-like filaments on the carapace is a remarkable feature among the 25 species of *Gastrosaccus* known to date, although the latter character is shared by several species of *Gastrosaccus* as well as *Eurobowmaniella* Murano, 1995 (see Bamber and Morton 2012). Interestingly, *Chlamydopleon* Ortmann, 1893 has a similar type of articulated projection on the fifth abdominal somite. Based on this and several other congruent characters, *Bowmaniella* sensu Băcescu, 1968 is now split into the two genera, *Chlamydopleon* Ortmann, 1893 and *Coifmanniella* Heard & Price, 2006 (Wittmann 2009). In the “*Gastrosaccus sorrentoensis*” species group, this articulated process may be present throughout the entire life history (Bamber and Morton 2012, present study).

*Iiella kojimaensis* (Nakazawa, 1910) has been believed to possess a posterodorsal process on the fifth somite (Ii 1964, Băcescu and Udrescu 1982, Bamber and Morton 2012). This morphological recognition in *Iiella kojimaensis* is probably due to Ii (1964: 240), who noted that the fifth abdominal somite of this species has an obtuse posterodorsal projection. That projection suggested by Ii, however, might have been confused with a transverse ridge (fold/apophysis) situated at the anterior dorsal end of the sixth somite (cf., Jo 1991, personal observations), which is a structure commonly observed across species of *Gastrosaccus* and its closer allies (e.g., Jo 1991, Jo and Murano 1992, Murano 1995, Hanamura 1997, personal observations).

Similarly, *Eurobowmaniella simulans* (Tattersall, 1915) has been reported to bear a mid-dorsal apophysis on the fifth abdominal somite in its early life stage. We have re-examined specimens of *Eurobowmaniella simulans* (TL 2.3−7.1 mm) collected from the northern Malacca Strait, but have failed to observe an articulated process, even in the smallest specimens. However, an anterodorsal ridge of the sixth abdominal somite is present throughout the observed size ranges (see also Hanamura et al. 2007). Further our examination demonstrated that smallest juveniles of *Eurobowmaniella simulans* (TL 2.5 mm) have a smooth posterodosal carapace edge, where the spine-like filaments subsequently appeared in later stages (TL ≥ 3 mm). Bamber and Morton (2012) remarked that the posterodorsal filaments of the carapace observed in *Gastrosaccus sorrentoensis* are also fewer in numbers in smaller individuals than in fully grown adults, indicating its progressive development with growth.

The East Australian species *Haplostylus brisbanensis* (Băcescu & Udrescu, 1982) appears to have the same type of articulated process that is found in *Chlamydopleon* and the “*Gastrosaccus sorrentoensis*” species group. *Haplostylus brisbanensis*, however, has no spine-like filaments on the carapace and the endopod of the third male pleopod is reduced to a non-articulated lobe, a typical form for *Haplostylus* (see Băcescu and Udrescu 1982, Wooldridge and McLachlan 1986).

The character distribution among the genera of *Gastrosaccus* affinities is an admixture of character states (Table 2). The articulated abdominal process is shared by small members of this animal group that are found in the geographically isolated Pacific and Atlantic coasts of American continents and Indonesia-Western Australian coasts. This remarkable morphological structure is most probable to have been acquired independently in different lineages rather than it is regarded as a plesiomorphic character and lost in the majority of gastrosaccini descents, although its phylogenetic significance is still not very clear. The taxonomic position of the Indonesia–Australian species of *Gastrosaccus* (and also *Haplostylus brisbanensis*) that bear the articulated abdominal process would be a subject of future consideration.

**Table 2.** Comparison between *Gastrosaccus* and related genera.

Item/genera	*Archaeomysis* Czerniavsky, 1882	*Chlamydopleon* Ortmann, 1893	*Coifmanniella* Heard & Price, 2006	*Eurobowmaniella* Murano, 1995	*Gastrosaccus* Norman, 1892	*Haplostylus* Kossman, 1888	*Iiella* Băcescu, 1968
Posterior dorsal margin of carapcace	no median concavity; no fringe of spine-like filaments	median concavity and protruded median lobes; no fringe of spine-like filaments but with minute undulations	median concavity and protruded medianlobes; no fringe of spine-like filaments but minute undulations	no median concavity; fringe of spine-like filaments	median concavity and protruded median lobes present/absent; fringe of spine-like filaments present or absent	median concavity and protruded median lobes present/absent; no fringe of spine-like filaments	no median concavity; no fringe of spine-like filaments
Labrum	single long anteromedian spine	long anteromedian and several additional smaller spines	long anteromedian and several additional smaller spines	long anteromedian and several additional smaller spines	single long anteromedian spine	single long anteromedian spine	long anteromedian and several additional smaller spines
Abdomen (fifth somite)	no movable process at posterodorsal margin	movable process present at posterodorsal margin	no movable process at posterodorsal margin	no movable process at posterodorsal margin	no movable process at posterodorsal margin (except *Gastrosaccus sorrentoensis* and its closest affinities)	no movable process at posterodorsal margin (except H. brisbanensis)	no movable process at posterodorsal margin
Male second pleopod	exopod multi-segments; endopod multi-segments	exopod multi-segments; endopod uni-segment	exopod multi-segments; endopod uni-segment	exopod multi-segments; endopod multi-segments	exopod multi-segments; endopod multi-segments	exopod multi-segments; endopod uni-/multi-segments	exopod multi-segments; endopod multi-segments
Male third pleopod	exopod styliform; endopod multi-segment	exopod greatly complicated; endopod uni-segment	exopod greatly complicated; endopod uni-segment	exopod moderately modified; endopod multi-segments	exopod styliform; endopod multi-segments	exopod styliform; endopod uni-segment	exopod styliform; endopod multi-segments
Female pleopods	first pleopod biramous; second-fifth pleopods with rudimentary exopod	first pleopod biramous; second-fifth pleopods rudimentary rod-shaped lobe	first pleopod biramous; second-fifth pleopods rudimentary rod-shaped lobe	first pleopod biramous; second-fifth pleopods rudimentary rod-shaped lobe	first pleopod biramous; second-fifth pleopods rudimentary rod-shaped lobe	first pleopod biramous; second-fifth pleopods rudimentary rod-shaped lobe	first pleopod uniramous; second-fifth pleopods rudimentary rod-shaped lobe
Geographical range	North Pacific, from Hong Kong to California, through subarctic Pacific islands	tropical and subtropical coasts of East Pacific as well as West Atlantic	tropical coasts of East Pacific and tropical and subtropical coasts of West Atlantic	Northeastern Indian Ocean, from India to northwestern Malay Peninsula	tenperate to tropical waters of East Atlantic through Indian Ocean to Australia and New Zealand	Mediterranean Sea through Indian Ocean to western Pacific	Northwestern Pacific, from Singapore to Japan and Korea

Data sources: Jo and Murano (1992), Murano (1995), Hanamura (1997), Heard and Price (2006), Wittmann (2009), Fukuoka (2011), and Bamber and Morton (2012).

The finding of *Gastrosaccus lombokiensis*, as a third species of the “*Gastrosaccus sorrentoensis*” group, implies possible further diversification of this species group in the eastern Indian Ocean. Despite a rather intensive survey of the beach mysids across the South East Asia, other species of gastrosaccini mysids could not be found except for *Eurobowmaniella simulans*, which was sampled in sandy beaches of Langkawi Island, off the north-east coast of Peninsular Malaysia (Hanamura et al. 2007). Hence, the “*Gastrosaccus sorrentoensis*” species group is highly probable to be endemic to the south-east Indian Ocean Arc, in a somewhat restricted geographical area from Indonesia through Australia.

### Key to the species of the “*Gastrosaccus sorrentoensis*” group

**Table:** 

1	Sharp projection absent at proximal ventral part of telson, apical telson seta very slightly longer than penultimate one	*Gastrosaccus yuyu* Bamber & Morton, 2012
–	Sharp projection present at proximal ventral part of telson, apical telson seta distinctly longer than penultimate one	2
2	Posteromedian margin of carapace with as many as 25 spine-like filaments; endopod of eighth thoracic limb composed of 14−16 segments; basal article of exopod of male third pleopod divided into 2 or 3 sub-segments; telson with pigmented spots at inner side of fifth lateral setae in female	*Gastrosaccus sorrentoensis* Wooldridge & McLachalan, 1998
–	Posteromedian margin of carapace with less than 12 spine-like filaments; endopod of eighth thoracic limb composed of 8 or 9 segments; basal article of exopod of male third pleopod without distinct sub-segments; telson with melanophore widely spread along margins in both sexes	*Gastrosaccus lombokiensis* sp. n.

## Supplementary Material

XML Treatment for Gastrosaccus

XML Treatment for Gastrosaccus lombokiensis
